# Caveolin-1-Mediated Cholesterol Accumulation Contributes to Exaggerated mGluR-Dependent Long-Term Depression and Impaired Cognition in *Fmr1* Knockout Mice

**DOI:** 10.1007/s12035-023-03269-z

**Published:** 2023-03-01

**Authors:** Li Luo, Le Yang, Kun Zhang, Shi-Meng Zhou, Yan Wang, Liu-Kun Yang, Bin Feng, Shui-Bing Liu, Yu-Mei Wu, Ming-Gao Zhao, Qi Yang

**Affiliations:** 1 Precision Pharmacy & Drug Development Center, Department of Pharmacy, Tangdu Hospital, Fourth Military Medical University, Xi’an, 710038 China; 2 Department of Pharmacology, School of Pharmacy, Fourth Military Medical University, Xi’an, 710032 China; 3 Department of Gastroenterology and Endoscopy Center, Fourth Military Medical University, No.986 Hospital, Xi’an, 710054 China; 4 State Key Laboratory of Military Stomatology, Department of Pharmacy, School of Stomatology, National Clinical Research Center for Oral Diseases, Shaanxi International Joint Research Center for Oral Diseases, Fourth Military Medical University, Xi’an, 710054 China

**Keywords:** Metabotropic glutamate receptor, Long-term depression, Caveolin-l, Endocytosis, Cholesterol, Fragile X syndrome

## Abstract

Fragile X syndrome (FXS) is one of the most common inherited mental retardation diseases and is caused by the loss of fragile X mental retardation protein (FMRP) expression. The metabotropic glutamate receptor (mGluR) theory of FXS states that enhanced mGluR-dependent long-term depression (LTD) due to FMRP loss is involved in aberrant synaptic plasticity and autistic-like behaviors, but little is known about the underlying molecular mechanism. Here, we found that only hippocampal mGluR-LTD was exaggerated in adolescent *Fmr1* KO mice, while N-methyl-D-aspartate receptor (NMDAR)-LTD was intact in mice of all ages. This development-dependent alteration was related to the differential expression of caveolin-1 (Cav1), which is essential for caveolae formation. Knockdown of Cav1 restored the enhanced mGluR-LTD in *Fmr1* KO mice. Moreover, hippocampal Cav1 expression in *Fmr1* KO mice induced excessive endocytosis of the α-amino-3-hydroxyl-5-methyl-4-isoxazolepropionate (AMPA) receptor subunit GluA2. This process relied on mGluR1/5 activation rather than NMDAR. Interference with Cav1 expression reversed these changes. Furthermore, massive cholesterol accumulation contributed to redundant caveolae formation, which provided the platform for mGluR-triggered Cav1 coupling to GluA2. Importantly, injection of the cholesterol scavenger methyl-β-cyclodextrin (Mβ-CD) recovered AMPA receptor trafficking and markedly alleviated hyperactivity, hippocampus-dependent fear memory, and spatial memory defects in *Fmr1* KO mice. Together, our findings elucidate the important role of Cav1 in mediating mGluR-LTD enhancement and further inducing AMPA receptor endocytosis and suggest that cholesterol depletion by Mβ-CD during caveolae formation may be a novel and safe strategy to treat FXS.

**Supplementary Information:**

The online version contains supplementary material available at 10.1007/s12035-023-03269-z.

## Introduction

Fragile X syndrome (FXS) is the most common inherited neurodevelopmental disease and is characterized by intellectual disability, hyperactivity, attention deficits, and autistic-like behaviors [1]. It is caused by the full mutation of the *Fmr1* gene, which is characterized by the excessive expansion of CGG trinucleotide repeats (≥ 200) in the 5′ untranslated region (UTR) of the gene. These expanded CGG triplet repeats are hypermethylated with consequent transcriptional gene silencing, halting gene expression, thereby resulting in a reduction or absence of fragile X mental retardation protein (FMRP) [2, 3]. With the development of *Fmr1* knockout (KO) mice [4], numerous animal studies have attempted to uncover the pathology of FXS. Over the last few years, the metabotropic glutamate receptor (mGluR) theory of FXS has gained much support [5]. The theory states that α-amino-3-hydroxyl-5-methyl-4-isoxazolepropionate (AMPA) receptor internalization, triggered by mGluR5 stimulation, is exaggerated in *Fmr1* KO mice and associated with protein synthesis, accounting for the enhanced hippocampal long-term depression (LTD) [5, 6]. Although loss of FMRP leads to prolonged mGluR signaling, little is known about the developmental alterations in experience-dependent synaptic function. The molecular mechanisms underlying these changes have yet to be elucidated, but are thought to be related to the role of FMRP in mRNA transport and translation [7–10].

We previously reported that *caveolin-1 (Cav1)* is a targeted mRNA repressed by FMRP [11]. Cav1 is the best-characterized mediator of clathrin-independent endocytosis and can mediate rapid endocytosis [12, 13]. Within hippocampal neurons, Cav1 has been found to bind and colocalize with Group I mGluRs [14]. Research in *Cav1* KO mice suggests that Cav1 is required for normal coupling of mGluR1/5 to downstream signaling cascades and induction of mGluR-LTD [15]. Due to the important role of Cav1 in cholesterol transport and homeostasis, findings suggest that altered cholesterol homeostasis may affect Cav1 expression and the pathogenesis of several neurodegenerative diseases [16]. However, the role of Cav1 regulation via cholesterol in exaggerated mGluR-LTD due to lack of FMRP is not known.

Here, we report that exaggerated mGluR-LTD in the *Fmr1* KO hippocampus is development-dependent and caused by overexpressed Cav1, in accordance with the loss of FMRP. We show that under mGluR1/5 activation, Cav1 facilitates AMPA receptor endocytosis. Knockdown of excess Cav1 alleviates aberrant mGluR-LTD and AMPA receptor endocytosis. Moreover, massive cholesterol accumulation in neurons provides the structural basis for caveolae hyperfunction. Upon depletion of cellular cholesterol with methyl-β-cyclodextrin (Mβ-CD), learning and memory disorders in *Fmr1* KO mice are significantly relieved. Together, these findings provide evidence for an unknown molecular mechanism of the mGluR theory in FXS and suggest that Mβ-CD may be a potential drug to treat FXS.

## Materials and Methods

### Animals

We used male *Fmr1* KO (Stock No: 004624, The Jackson Laboratory, Bar Harbor, ME, USA), *Fmr1* WT (Stock No: 004828, The Jackson Laboratory), and, as controls, C57BL/6 J mice for this study. The mice were housed on a 12-h light/dark cycle and provided ad libitum access to food and water. All procedures involving animals were carried out in accordance with the guidelines of the Fourth Military Medical University.

### Drugs and Antibodies

(RS)-3,5-DHPG and dynasore were purchased from Tocris Bioscience (Bristol, UK). AP5 (D-2-amino-5-phosphonopentanoate), MPEP, cholesterol, and methyl-β-cyclodextrin (Mβ-CD) were obtained from Sigma (St. Louis, MO, USA). We used the following antibodies: anti-FMRP (MAB2160; Millipore, Billerica, MA, USA), anti-Cav1 (ab2910, Abcam, Cambridge, UK), anti-GluA1 (ab31232; Abcam), anti-GluA2 (ab20673; Abcam), anti-pan-cadherin (C1821; Sigma), anti-ERK1/2 (sc514302; Santa Cruz Biotechnology, Santa Cruz, CA, USA), anti-pERK1/2 (sc81492; Santa Cruz Biotechnology), anti-mTOR (sc517464; Santa Cruz Biotechnology), anti-pmTOR (sc293133; Santa Cruz Biotechnology), and anti-β-actin (A5316; Sigma).

### DNA Constructs and Lentiviral Vector

We obtained full-length mouse *Cav1* by reverse transcription–polymerase chain reaction (RT‒PCR) using the following primers: 5′-CCCTCGAGGGAAACCTCCTCAGAGCC-3′ (forward) and 5′-CGGAATTCAAATTTGCTGCTGCGAGA-3′ (reverse). The *Cav1* cDNA was cloned into a lentiviral expression vector provided by GenePharma (Shanghai, China). To knock down *Cav1*, two sequences were targeted: AAGGAGATTGACCTGGTCAAC (*Cav1* shRNA-1) and GCAAGATATTCAGCAACATCC (*Cav1* shRNA-2). All lentiviral vectors were carried with GFP tags, and the infected cells could be identified under a fluorescence microscope. According to the manufacturer’s instructions, animals and cells were treated with varying doses of the vector (1 × 10^9^ pfu/ml) for 5–7 days. Mice were anesthetized with pentobarbital sodium, and *Cav1* shRNA lentivirus was stereotaxically microinjected into the bilateral CA1 region of the hippocampus (2.3 mm anterior to bregma, ± 1.5 mm lateral to midline, and 1.7 mm ventral to bregma) at a rate of 0.1 µl/min for 10 min, resulting in a dose of 1 µl of lentivirus per side. One week after surgery, hippocampal slices were prepared from the animals.

### Hippocampal Slice Preparation

Hippocampal slices from male WT and *Fmr1* KO mice were prepared as previously described [17]. Two different age groups were used: p8-p15 and p28-p35. Slices were cut in oxygenated solution (in mM): 250 sucrose, 2.5 KCl, 0.5 CaCl_2_, 6 MgSO_4_, 1.2 NaH_2_PO_4_, 25 NaHCO_3_, and 10 D-glucose. After cutting, the slices recovered at 34 °C in aCSF (in mM) as follows: 124 NaCl, 4.4 KCl, 2 CaCl_2_, 1 MgSO_4_, 1 NaH_2_PO_4_, 25 NaHCO_3_, and 10 D-glucose. After 10 min, the slices were placed in aCSF at room temperature for an additional 1–2 h and gassed with 95% O_2_–5% CO_2_.

### Field Electrophysiological Recordings

Briefly, individual slices were placed in an MED64 probe (MED-P515A, 8 × 8 array; interpolar distance, 150 µm) positioned on the Schaffer collateral-CA1 pathway of the dorsal hippocampus. Stable baseline responses were recorded for at least 1 h, and slices were treated with DHPG (100 μM) for 10 min or low-frequency stimulation (LFS; 900 pulses at 1 Hz for 15 min). After the wash period, mGluR-dependent LTD was induced by application of DHPG, and NMDAR-dependent LTD was induced by LFS. It has been reported that some LFS-induced LTD is also sensitive to mGluRs [18]. Therefore, the effect of LFS on inducing LTD in hippocampal slices from WT or KO mice was examined in the presence or absence of the mGluR5 antagonist MPEP (40 μM). Similarly, for mGluR-dependent LTD, NMDAR antagonist AP5 (50 μM) was applied to determine whether there was any effect on DHPG-induced LTD. Multichannel electrophysiological data were collected every 1 min, and fEPSP slopes were measured and analyzed using Mobius software (Panasonic Alpha-Med Sciences, Tokyo, Japan) [11, 19].

### Input/Output Curve Plotting

To determine whether *Cav1* shRNA had an effect on the baseline responses recorded from hippocampal field potentials in *Fmr1* KO mice, input/output curves were plotted. The X-axis represents different stimulation intensities. The slope of the fEPSP under each stimulus intensity was measured, and I/O curves were generated.

### Primary Neuronal Culture

Neuronal hippocampal cultures were prepared from embryonic 17-day-old (E17) mice and grown in neurobasal medium supplemented with B27 and 10% fetal bovine serum (FBS), as described previously [20]. The cultures were infected with lentivirus at 6 days in vitro (DIV) or treated with drugs at 10 DIV.

### Caveolae Membrane Fraction Purification

Caveolae-enriched membrane fractions were purified by using a Caveolae/Rafts Isolation Kit (CS0750; Sigma). According to the manufacturer’s instructions, cells grown on 10-cm plates were washed in cold PBS and scraped into 1-ml lysis buffer containing 1% Triton X-100. Following homogenization and sonication, 0.84 ml of the suspension was adjusted to 35% OptiPrep by adding 1.16 ml of OptiPrep density gradient medium and placed at the bottom of an ultracentrifuge tube. A discontinuous gradient was generated by layering 2 ml of 30%, 25%, 20%, and 0% OptiPrep (diluted in lysis buffer) in decreasing order. The gradients were centrifuged at 200,000 × *g* using an SW41Ti rotor (Beckman Coulter, Palo Alto, CA, USA) for 4 h at 4 °C. From the top of each gradient, ten equal fractions (1 ml) were collected. A light scattering band was observed at fractions 4–5, corresponding to caveolae [21]. Each fraction was concentrated by using a Pierce® SDS‒PAGE Sample Prep Kit (No. 89888; Thermo Scientific, Waltham, MA, USA) and analyzed by immunoblotting.

### Synaptosome Preparation

Synaptosomes were prepared according to the protocol described by Zhou et al*.* [22]*.* Briefly, the homogenates of neuronal cells were diluted with a discontinuous Percoll gradient (P4937; Sigma). After centrifugation for 5 min at 33,000 × *g* at 4 °C, the layers between 10 and 20% Percoll were collected, washed in HEPES-buffered medium (140 mM NaCl, 5 mM KCl, 5 mM NaHCO_3_, 1.2 mM NaH_2_PO_4_, 1 mM MgCl_2_, 10 mM glucose, 10 mM HEPES, pH 7.4) and further centrifuged at 22,000 × *g* for 15 min at 4 °C. The pellet was resuspended in Krebs–Ringer buffer and recentrifuged at 2000 × *g* for 15 min. The final pellet was resuspended in 100 μl of neuronal lysis buffer and immediately used for Western blot analysis.

### Surface Biotinylation Assay

Surface AMPA receptors were detected by biotinylation assay as described previously [23]. After treatment, neurons were incubated with 1 mg/ml sulfo-NHS-LC-biotin (No. 21335; Thermo Scientific) in aCSF for 30 min on ice, and then the reaction was stopped by removal of the solution and incubation in 100 mM ice-cold glycine in aCSF for 20 min. After cell lysis with RIPA buffer, biotinylated surface (membrane) proteins were precipitated with 30 μl of Dynabead Biotin Binder (No. 11047; Thermo Scientific), and the residual samples were cytoplasm. Both membrane and cytosolic samples were subjected to SDS‒PAGE and probed with anti-GluA C-terminal antibodies. Pan-cadherin and β-actin were used as internal controls.

### Immunoblot Analysis

Samples were separated using a 12% SDS‒PAGE gel and transferred to PVDF membranes (Millipore, Billerica, MA, USA). The membranes were blocked in PBS Tween-20 (1%) containing 5% BSA and incubated with primary antibodies overnight at 4 °C. The primary antibodies were visualized using secondary antibodies conjugated to horseradish peroxidase (Santa Cruz Biotechnology) and ECL reagent (GE Healthcare Pharmacia, Uppsala, Sweden). The densitometric analysis of bands was conducted using a ChemiDoc XRS (Bio-Rad, Hercules, CA, USA) and quantified using Quantity One version 4.1.0 (Bio-Rad).

### Cholesterol Detection

The cellular lipid content was extracted from aliquots of the hippocampus or serum. Both total and free cholesterol concentrations were measured using a Cholesterol Quantitation Kit (MAK043; Sigma) according to the manufacturer’s directions [24]. Cholesterol was quantified by densitometry and comparison with cholesterol standards run on the same plate. The measurements were performed by a GloMax® Discover Multimode Microplate Reader (Promega). For cholesterol staining, hippocampal sections were labeled with filipin III, 50 μg/ml, for 2 h in the dark. To focus on the cholesterol levels in neuronal cells, permeabilized sections were also incubated with monoclonal NeuN (ab177487; Abcam) overnight at 4 °C and then with a FITC-conjugated secondary antibody for 40 min at room temperature. Images were acquired with a living cell microscopic imaging station (BioTek, Cytation 1, USA).

### Behavioral Tests

Five-week-old KO mice were divided into four groups and subcutaneously injected with 125, 250, or 500 mg/kg Mβ-CD or saline twice per week for 2 weeks. Each group contained 10 mice. All mice were brought into the testing room 2 h before the tests. Behavior was assessed by an experimenter blinded to the treatment. All behavioral tests were performed during the light period on the designated days of the experiment.

### Open-Field Test

Each animal was placed in the center of a Plexiglas box (30 × 30 × 30 cm^3^) and allowed to freely explore for 15 min. The total distance traveled and time spent in the central area were analyzed using a video-tracking system (MedAssociates, St. Albans, VT, USA).

### Fear Conditioning Test

On the training day (day 1), mice were placed in a dark chamber and presented with three tone (conditioned stimulus (CS): 90 dB, 5 kHz)-shock (unconditioned stimulus (US): 2.0 s, 1.0 mA) pairings with a varying intertrial interval of 30–90 s. On day 2, the mice were re-exposed to the fear conditioning chamber for 8 min to test contextual fear recall, and freezing time was averaged across the entire session. On day 3, the mice were tested for cued fear recall. The session started with a 3-min acclimation period; then, 10 blocks of five CS were presented for 30 s each with an intertrial interval of 5 s, and freezing was recorded during each presentation. For analysis, total freezing was averaged across all CS presentations. Freezing was determined using analysis software (Med Associates, San Diego, CA, USA) [25].

### Morris Water Maze Test

A circular tank (120 cm in diameter and 50 cm in height) with nontoxic tempera paint solution (maintained at 22–25 °C) was used. Visual cues were placed in the four corners for spatial orientation. All mice were trained for 4 d (60 s trial time, 4 trials each day) to find a hidden platform in the Morris water maze. One hour after the final learning trial, a single 60-s probe trial was conducted without the platform. During the experiment, the entry quadrant varied, but the platform location remained constant. The latency to enter the target area (the previous platform location), number of platform crossings, and swimming speed were calculated [26].

### Statistical Analyses

The sample size was chosen based on previous publications. Based on the number of comparisons and the pattern of data distribution, appropriate statistical tests were used to analyze the data. Data analysis was conducted using Prism 8 software (GraphPad, San Diego, CA, USA). The data are expressed as the means ± SEMs for at least three independent experiments. For two-group comparisons, statistical significance was determined using unpaired two-tailed Student’s *t* test. Multigroup analyses were performed using one/two-way ANOVA. Significance was defined as *p < 0.05; **p < 0.01.* P* values < 0.05 were considered to indicate statistical significance. Statistical analyses for every experiment are described accordingly in the figure captions.

## Results

### *Fmr1* KO Mice Exhibit Altered mGluR-LTD but Intact NMDAR-LTD

**Fig. 1:**
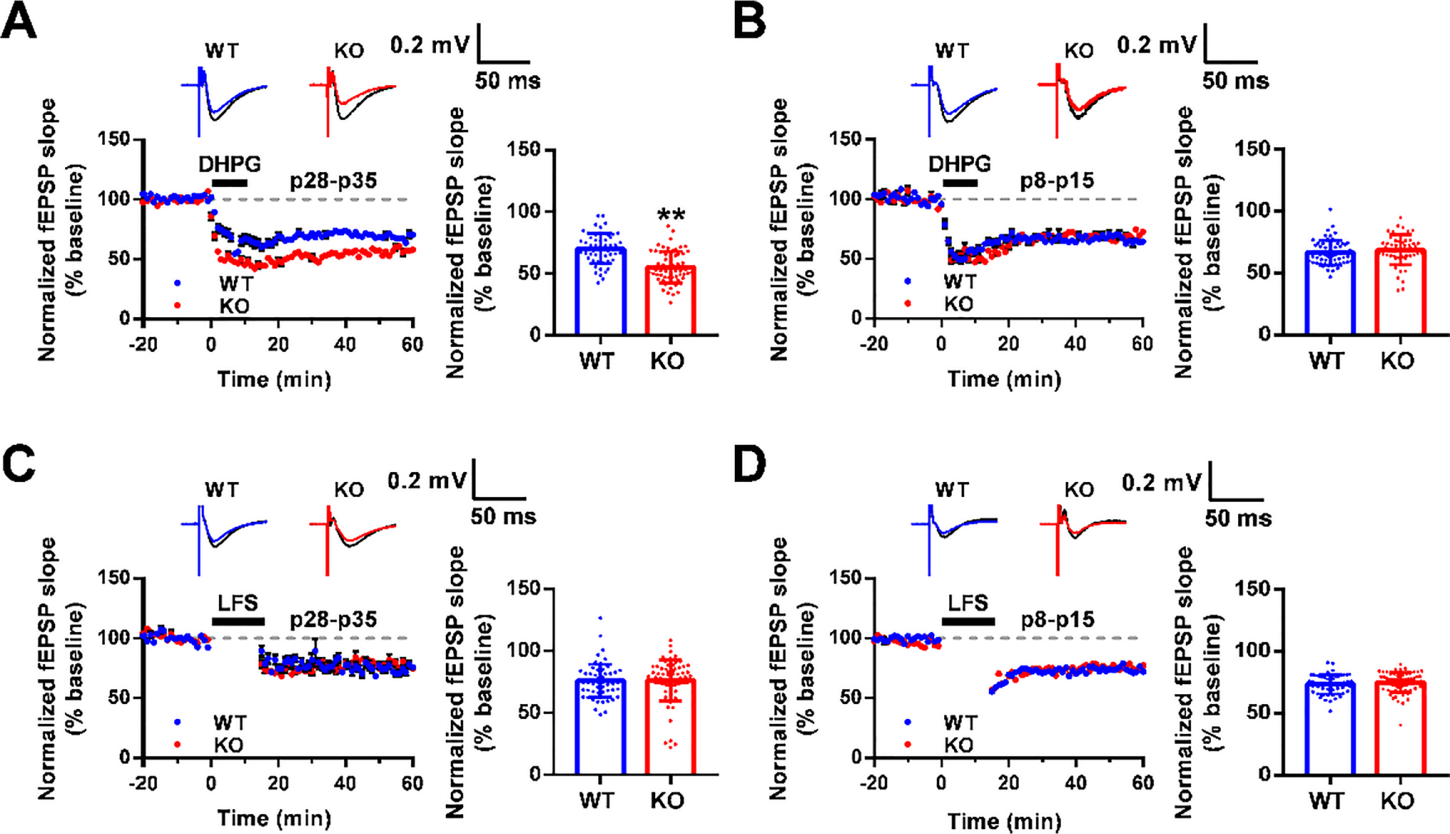
Enhanced mGluR-LTD in the adolescent *Fmr1* KO hippocampus. **A** Induction of mGluR-LTD by bath application of DHPG (100 μM, 10 min) at age p28-p35 in WT and *Fmr1* KO mice. *n* = 56 channels recorded from 8 slices of five WT mice, *n* = 63 channels recorded from 9 slices of six KO mice; ** *p* < 0.01 versus WT mice, unpaired two-tailed Student’s *t* test. **B** DHPG induced mGluR-LTD at age p8-p15 in WT and *Fmr1* KO mice. *n* = 58 channels recorded from 8 slices of five WT mice, *n* = 53 channels recorded from 9 slices of five KO mice, unpaired two-tailed Student’s *t* test. **C** NMDAR-LTD was induced by LFS (900 pulses at 1 Hz, 15 min) at age p28-p35 in WT and *Fmr1* KO mice.* n* = 61 channels recorded from 9 slices of five WT mice, *n* = 73 channels recorded from 9 slices of six KO mice, unpaired two-tailed Student’s *t* test. **D** LFS induced NMDAR-LTD at age p8-p15 in WT and *Fmr1* KO mice. *n* = 55 channels recorded from 7 slices of four WT mice, *n* = 69 channels recorded from 9 slices of five KO mice, unpaired two-tailed Student’s *t* test. Top, representative traces of 64 channels recorded at baseline (black) and 1 h after stimulation (colors). Calibration bars: 0.2 mV, 50 ms. Bottom left, normalized fEPSP slope (% baseline) of LTD from total active channels. Bottom right, summary graph of the final average fEPSP slope during the last 30 min

Based on the mGluR theory of FXS, exaggerated hippocampal mGluR-dependent LTD is a well-known characteristic in *fmr1* KO mice [5, 6]. To confirm these findings, we first recorded mGluR-LTD from the CA1 area in adolescent (p28-p35) mice. By using multichannel signals through a MED64 system, we recorded fEPSP slopes in the active channels from hippocampal slices. To induce pure mGluR-LTD, slices were preincubated with NMDAR antagonist AP5 for 30 min. Then, after perfusion with the selective mGluR Group I agonist DHPG for 10 min, synaptic responses were transiently reduced. When DHPG was washed out, the normalized fEPSP slope (% baseline) during the last 30 min between WT and KO mice (p28-p35) showed no significant change in the presence or absence of AP5** (**Fig. S1A, B). Next, we used DHPG to induce mGluR-LTD without AP5. After perfusion with DHPG for 10 min, synaptic responses were transiently reduced, and fEPSP slopes increased gradually in both WT and KO genotypes when DHPG was washed out. In WT mice, the normalized fEPSP slope (% baseline) of 56 channels during the last 30 min was averaged at 70.19 ± 1.62% (data from 8 slices of five mice), whereas the fEPSP slope of 63 channels in *Fmr1* KO mice was reduced to 55.11 ± 1.62% (data from 9 slices from six mice; Fig. 1A). Next, we checked the effect of DHPG on mGluR-LTD in neonatal (p8-p15) mice and found no detectable difference in baseline fEPSP slopes between WT and KO hippocampal slices (WT group, 66.61 ± 1.31% of 58 channels; KO group, 68.85 ± 1.67% of 53 channels; data from 8 slices of five WT mice and 9 slices of five KO mice; Fig. 1B), which is consistent with other reports [17].

It has been reported that some low-frequency stimulation (LFS)-induced LTD is also sensitive to mGluRs [18]. Therefore, we first explored whether the application of the mGluR5 antagonist MPEP had an effect on LFS-induced NMDAR-LTD. The results indicated that the normalized fEPSP slope (% baseline) of hippocampal slices from WT and KO mice (p28-p35) was not distinctly altered with or without MPEP (Fig. S1C, D). Thus, we further induced NMDAR-LTD by delivering LFS to Schaffer collateral axons in p8-p15 and p28-p35 mice without MPEP involvement. When recording from the CA1 area in p28-p35 mice, the normalized fEPSP slope (% baseline) from slices of WT and KO mice was averaged at 75.80 ± 1.72% (*n* = 61 channels recorded from 9 slices of five mice) and 76.34 ± 1.94% (*n* = 73 channels recorded from 9 slices of six mice; Fig. 1C), respectively. Similarly, in slices from p8-p15 mice, we found no significant difference in fEPSP slopes between the WT and KO groups (WT mice, 73.38 ± 1.04% of 55 channels; KO mice, 74.90 ± 0.96% of 69 channels; data from 7 slices of four WT mice and 9 slices of five KO mice; Fig. 1D). These results suggest that hippocampal NMDAR-LTD is intact in *Fmr1* KO mice. However, only at p28-p35 was enhanced mGluR-LTD observed in KO mice, suggesting that dysregulated mGluR activity in FXS is dependent on the specific developmental stage.

### Cav1 Translation Controlled by mGluR Is Dysregulated in *Fmr1* KO Mice

**Fig. 2:**
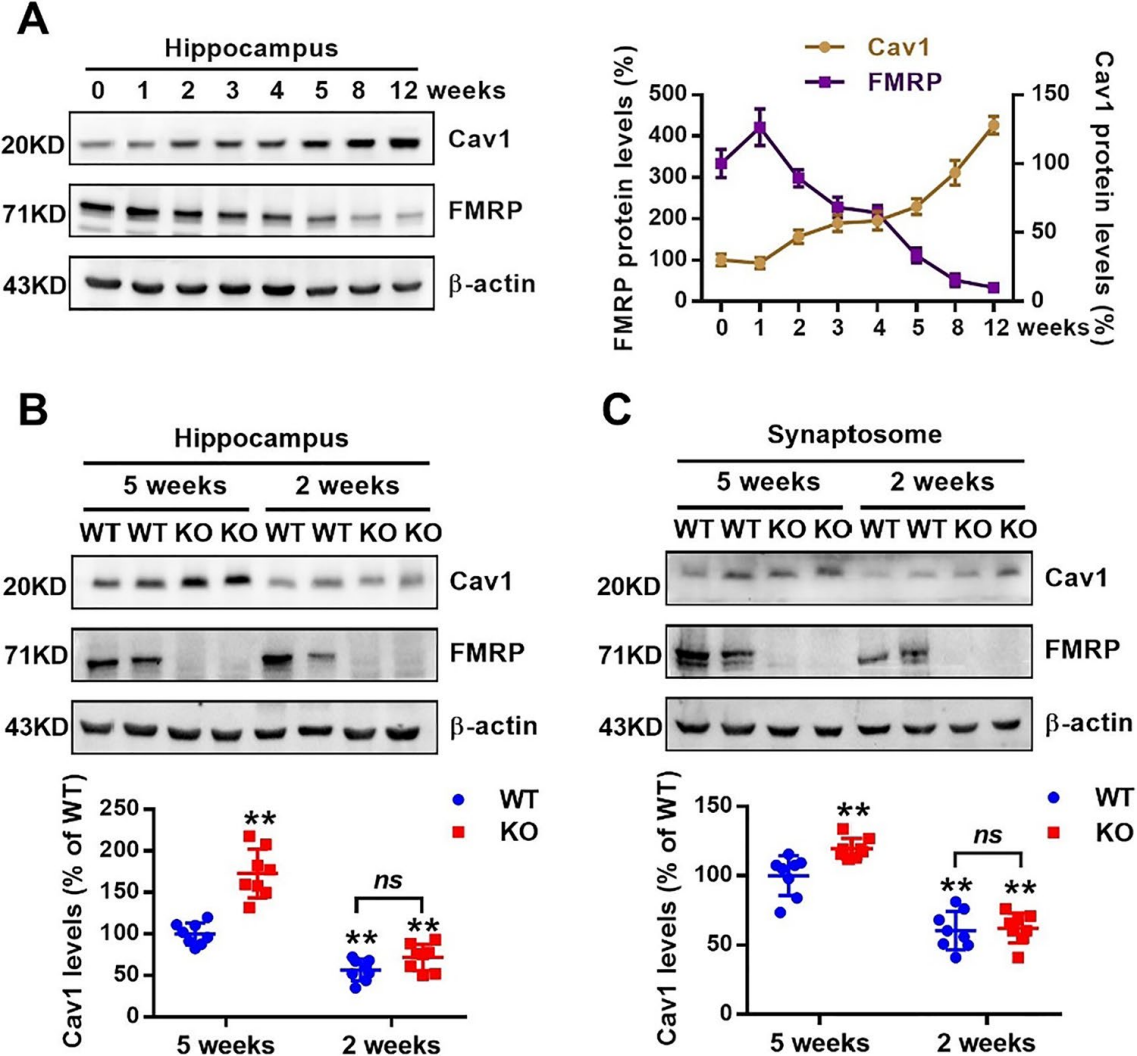
The expression of Cav1 is related to the level of FMRP. **A** Correlation between Cav1 and FMRP expression in the hippocampus at different weeks after birth. *n* = 8 mice per group. **B, C** Cav1 and FMRP protein levels were detected in hippocampus (**B**) and synaptosomes (**C**) from WT and *Fmr1* KO mice at 2 and 5 weeks. *n* = 8 mice per group; ** *p* < 0.01 versus WT mice at 5 weeks, n.s. represented the comparison between WT and KO mice at 2 weeks was not significant, two-way ANOVA with Tukey’s multiple comparisons test

Studies have reported that enhanced mGluR-LTD in *Fmr1* KO mice is due to exaggerated protein synthesis [5, 27]. In agreement with other studies [28], we found that FMRP levels in the mouse hippocampus were high in the first/second postnatal week and then gradually decreased (Fig. 2A). However, Cav1 expression gradually increased after birth and peaked when FMRP expression disappeared in WT mice (Fig. 2A). Previously, we demonstrated that FMRP interacted directly with *Cav1* mRNA [11]. Therefore, FMRP negatively regulates the translation of Cav1 during development. Furthermore, elevated Cav1 expression was observed only at 5 weeks in the KO hippocampus, while no difference was observed between WT and KO mice at 2 weeks (Fig. 2B). Similar results were observed in hippocampal synaptosomes (Fig. 2C). Thus, it can be seen that the age of the exaggerated enhancement of mGluR-LTD in the KO hippocampus keeps pace with the time period of the increase in Cav1 expression, indicating that increased Cav1 protein levels due to lack of FMRP since adolescence may underlie the exclusively enhanced mGluR-LTD at age p28-p35 in KO mice.

**Fig. 3:**
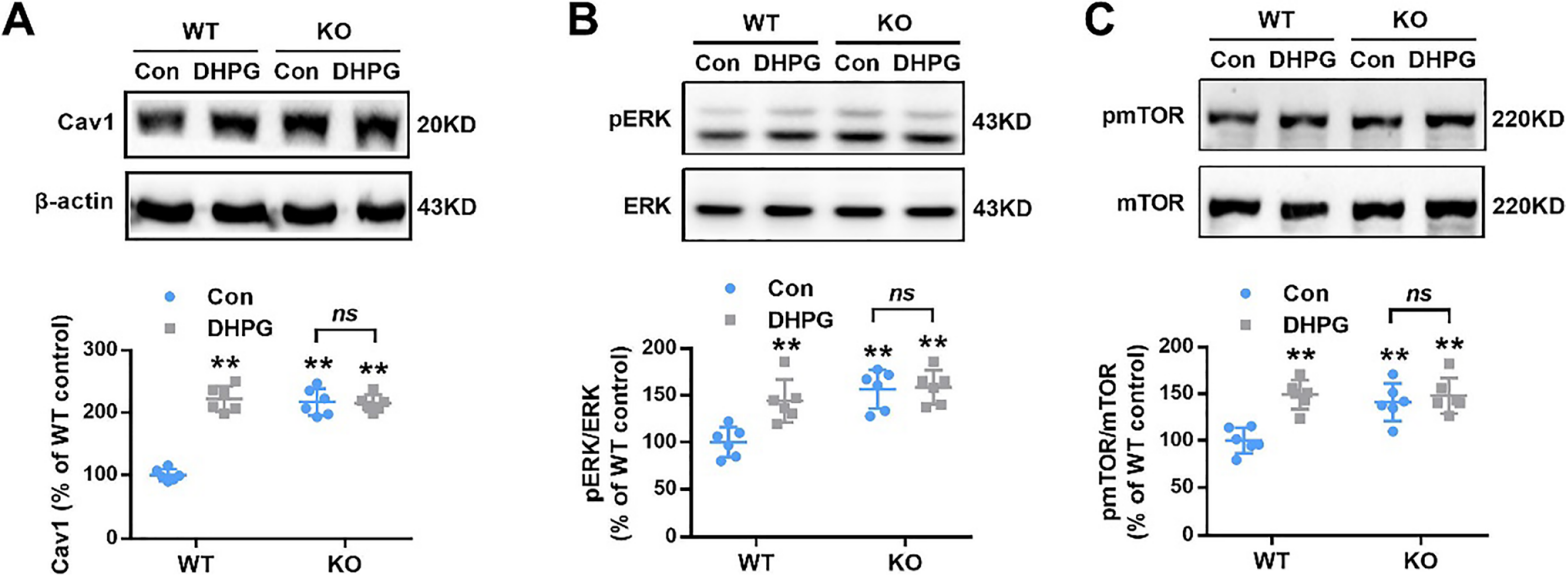
Effect of group I mGluR activation on Cav1, ERK, and mTOR expression. **A** DHPG (100 μM, 10 min) induced the expression of Cav1 in WT neurons (10 DIV), but led to no further increase in KO neurons.* n* = 6 dishes from three independent experiments; ** *p* < 0.01 versus WT control neurons, n.s. represented the comparison between KO control and DHPG was not significant, two-way ANOVA with Tukey’s multiple comparisons test. **B, C** Basal levels of phosphorylated ERK (**B**) and mTOR (**C**) were increased in *Fmr1* KO neurons. In contrast to WT neurons, there was no further DHPG (100 μM, 5 min)-induced phosphorylation of ERK (**B**) and mTOR (**C**) in KO neurons. *n* = 6 dishes from three independent experiments; ** *p* < 0.01 versus WT control, n.s. represented the comparison between KO control and DHPG was not significant, two-way ANOVA with Tukey’s multiple comparisons test

We then evaluated the effect of Group I mGluR activation on Cav1 translation. In the control group, Cav1 protein levels were higher in KO neurons than in WT neurons (Fig. 3A). However, DHPG induced a significant increase in the protein level of Cav1 in WT neurons but not in KO neurons (Fig. 3A). In addition, we also observed changes in mTOR and ERK phosphorylation levels by DHPG induction in WT and KO neurons, which were consistent with those of Cav1 (Fig. 3B, C). Takayasu et al. [15] reported that reduced mGluR-LTD in *Cav1 − / − *mice is protein synthesis-dependent and requires signaling via ERK and mTOR. Taken together with our finding, we considered Cav1 knockout would cause the elevation of basal phosphorylation of MEK and ERK1/2, but the regulatory mechanism was still unclear. These findings indicate that mGluR-dependent stimulation of Cav1 expression is regulated improperly in *Fmr1* KO mice.

### Aberrant mGluR-LTD and GluA2 Endocytosis Is Alleviated by Knocking Down Excessive Cav1

**Fig. 4:**
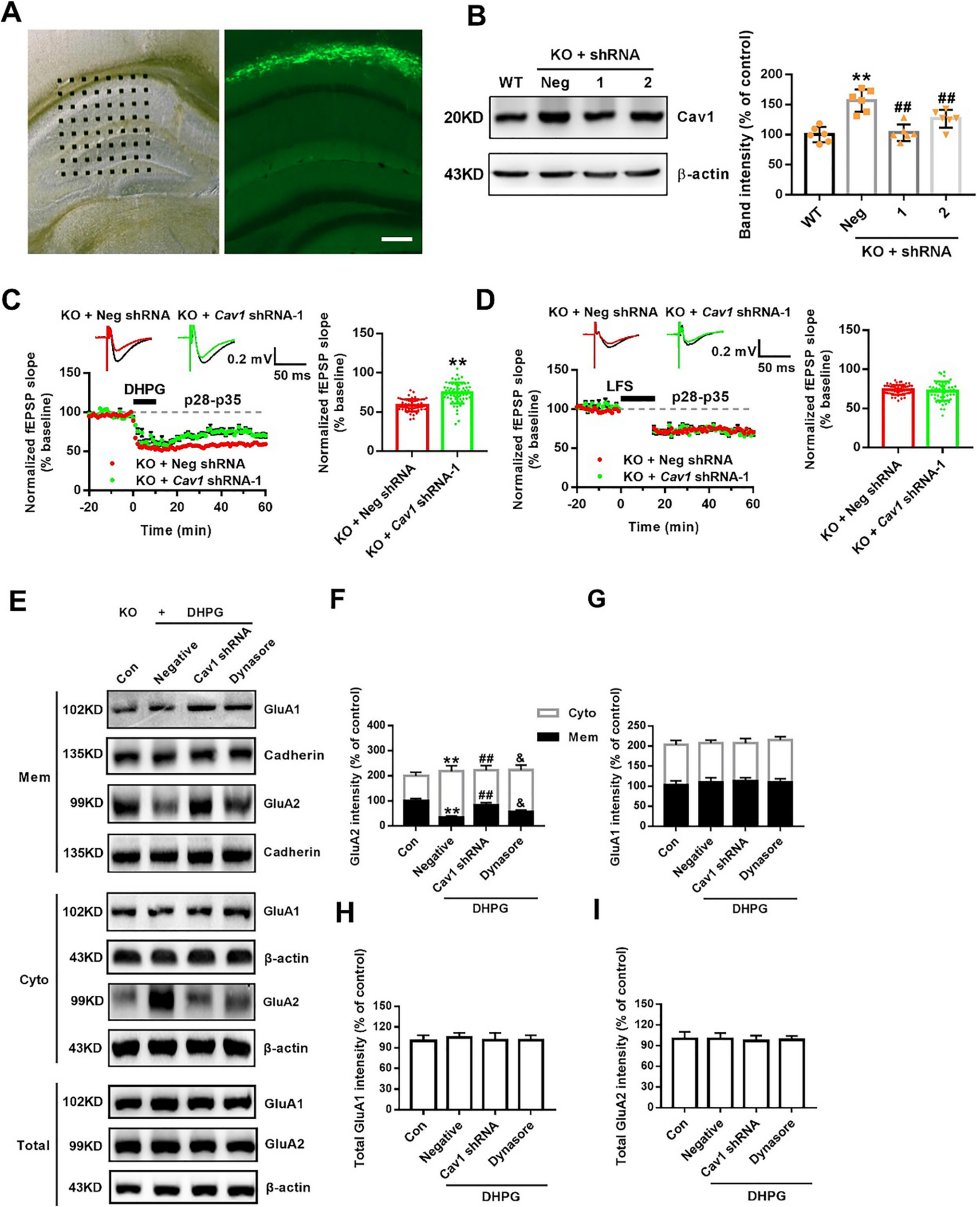
Knockdown of Cav1 rescues the aberrant mGluR-LTD and GluA2 endocytosis. **A** Schematic diagram showed the location of the Med64 probe on a hippocampal slice and the position of GFP-labeled lentivirus infection. Scale bar = 1000 μm. **B** Knockdown of Cav1 in the hippocampus detected after shRNA infection for 4 days. *n* = 6 mice per group; ** *p* < 0.01 versus WT mice; ## *p* < 0.01 versus negative KO mice, one-way ANOVA with Tukey’s multiple comparisons test. **C** After 7 days of infection, *Cav1* shRNA-1 decreased the normalized fEPSP slope of DHPG (100 μM, 10 min)-induced mGluR-LTD at age p28-p35 in KO mice.* n* = 58 channels recorded from 10 slices of six *Cav1* shRNA-1-infected mice, *n* = 59 channels recorded from 9 slices of six negative KO mice; ** *p* < 0.01 versus negative KO mice, unpaired two-tailed Student’s *t* test. **D** No significant differences in normalized fEPSP slope was observed in LFS (900 pulses at 1 Hz, 15 min)-induced NMDAR-LTD. *n* = 62 channels recorded from 9 slices of six *Cav1* shRNA-1-infected mice, *n* = 55 channels recorded from 9 slices of six negative KO mice, unpaired two-tailed Student’s t test. Top, representative traces of 64 channels recorded at baseline (black) and 1 h after stimulation (colors). Calibration bars: 0.2 mV, 50 ms. Bottom left, normalized fEPSP slope (% baseline) of LTD from total active channels. Bottom right, summary graph of the final average fEPSP slope during the last 30 min. **E** Representative Western blots showed differential distribution of GluA1/2 on membranes and in the cytoplasm by DHPG-treated *Cav1*-interfered KO neurons. **F, G** Quantitative analysis of cumulative western blot experiments. Immunoreactivities of GluA2 (**F**) and GluA1 (**G**) on membranes were normalized to cadherin, and the cytoplasm were normalized to β-actin.* n* = 6 dishes from three independent experiments. **H, I** Quantitative analysis of cumulative western blot experiments. Immunoreactivities of total GluA1 (**H**) and GluA2 (**I**) were normalized to β-actin.* n* = 6 dishes from three independent experiments; ** *p* < 0.01 versus control; ^##^ *p* < 0.01 versus negative + DHPG treatment; ^&^ *p* < 0.05 versus *Cav1*-shRNA + DHPG treatment, one-way ANOVA with Tukey’s multiple comparisons test

We next investigated whether dysregulated Cav1 induced by FMRP responded to augmented mGluR-LTD in *Fmr1* KO mice. Because Cav1 was overexpressed in the hippocampus of young KO mice, we microinjected GFP-labeled *Cav1* shRNA lentiviruses into the CA1 area to knock down Cav1 and found that *Cav1* shRNA-1 was the most effective (Fig. 4A, B). In the field potential recording, input/output curves showed that the fEPSP amplitude of each group increased as the stimulation intensity increased. However, there was no significant difference in the fEPSP slope between the *Cav1* shRNA-1 group and the negative control group, indicating that shRNA had no effect on the baseline responses (Fig. S2). We then induced LTD at age p28-p35 in the KO hippocampus after shRNA-1 lentivirus infection for 1 week. In DHPG-induced mGluR-LTD, compared with the negative controls, *Cav1* shRNA-1 significantly recovered the fEPSP slope of baseline (*Cav1* shRNA-1, 74.06 ± 1.74% of 59 channels; negative control, 58.56 ± 1.01% of 58 channels; data from 10 slices of six *Cav1* shRNA-1-infected mice and 9 slices of six negative control mice; Fig. 4C). However, no significant change was observed in LFS-induced NMDAR-LTD (*Cav1* shRNA-1, 72.11 ± 1.64% of 55 channels; negative control, 73.64 ± 0.77% of 62 channels; data from 9 slices of six *Cav1* shRNA-1-infected mice and 9 slices of six negative control mice; Fig. 4D). Furthermore, we wondered if inhibitory transmission may also be affected in these mice. Whole-cell patch recording in hippocampal neurons showed that inhibitory transmission in KO mice was normal and was not affected by *Cav1* shRNA-1 (Fig. S3). These data support a function for Cav1 in the over-induction of mGluR-LTD in *Fmr1* KO mice.

**Fig. 5:**
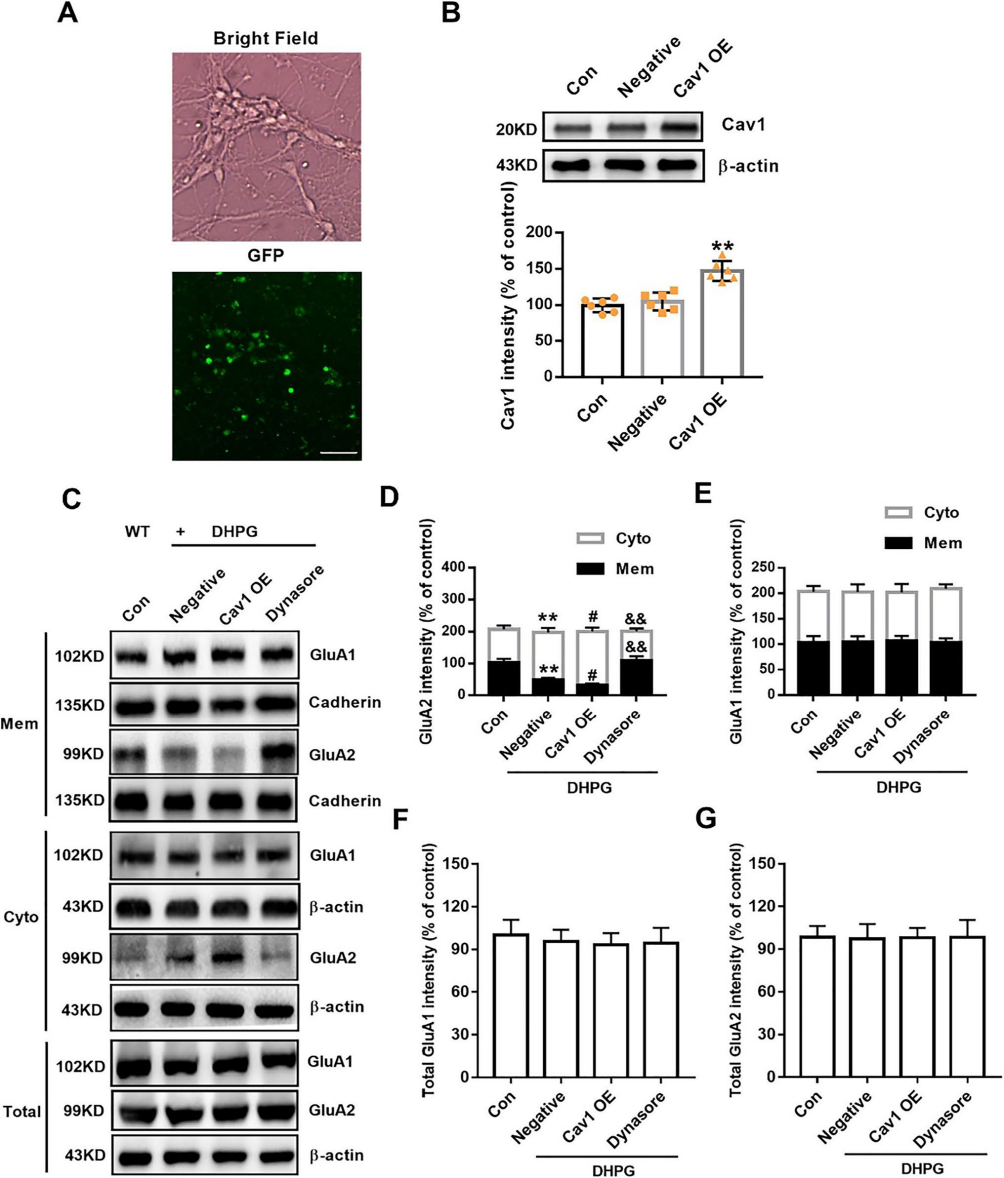
Overexpression of Cav1 regulates the GluA2 endocytosis induced by DHPG. **A** Bright-field image showing hippocampal neurons cultured in vitro for 10 days. GFP-positive neurons (green) indicate the successful infection of the lentivirus. Scale bar = 50 μm. **B** Cav1 expression was detected by Western blot after 4 days of infection. *n* = 6 dishes from three independent experiments; ** *p* < 0.01 versus negative control, one-way ANOVA with Tukey’s multiple comparisons test. **C** Representative western blots showed differential distribution of GluA1/2 on membranes and in the cytoplasm by DHPG-treated Cav1-OE WT neurons. **D, E** Quantitative analysis of cumulative western blot experiments. Immunoreactivities of GluA2 (**D**) and GluA1 (**E**) on membranes were normalized to cadherin, and the immunoreactivities in the cytoplasm were normalized to β-actin. *n* = 6 dishes from three independent experiments. **F, G** Quantitative analysis of cumulative western blot experiments. Immunoreactivities of total GluA1 (**F**) and GluA2 (**G**) were normalized to β-actin.* n* = 6 dishes from three independent experiments; ** *p* < 0.01 versus control; ^#^ *p* < 0.05 versus negative + DHPG treatment; ^&&^ *p* < 0.01 versus Cav1-OE + DHPG treatment, one-way ANOVA with Tukey’s multiple comparisons test

Given that the key event involved in LTD maintenance is internalization via endocytosis of synaptic AMPA glutamate receptors (AMPARs) [29–31], we evaluated the role of Cav1 in transporting the key subunits of AMPARs, GluA1 and GluA2. In cultured KO neurons, DHPG strongly promoted GluA2, but not GluA1, translocation from membranes to the cytoplasm (Fig. 4E). However, GluA2 expression on membranes induced by DHPG was increased after *Cav1* knockdown compared to the negative control conditions, although the distribution of GluA1 on membranes and cytoplasm was not altered (Fig. 4E–G). However, the change in GluA2 delivery was blocked by dynasore, an inhibitor of dynamin, which is a GTPase affecting membrane scission (Fig. 4E, F). In addition, there were no significant changes in GluA1 and GluA2 total protein levels (Fig. 4E, H, I). Conversely, we overexpressed Cav1 in cultured WT neurons (Fig. 5A, B) and found that Cav1 overexpression further decreased the membrane GluA2 level after DHPG incubation and that this effect was also blocked by dynasore (Fig. 5C, D). The GluA1 distribution in cell subregions was similarly unchanged (Fig. 5C, E). Also, the total protein levels of GluA1 and GluA2 showed no remarkable changes (Fig. 5C, F, G). Besides, Sucrose gradient fractions revealing membrane GluA2 localized in caveolae, identified by the presence of Cav1. DHPG treatment enhanced Cav1 expression and redistributed GluA2 from membrane rafts to the cytoplasm (Fig. S4). These results indicate that abnormal Cav1 assembles excessive GluA2 endocytosis in a dynamin-dependent manner, resulting in exaggerated mGluR-LTD in the KO hippocampus.

### Excessive Cholesterol Accumulated in *Fmr1* KO Mice

**Fig. 6:**
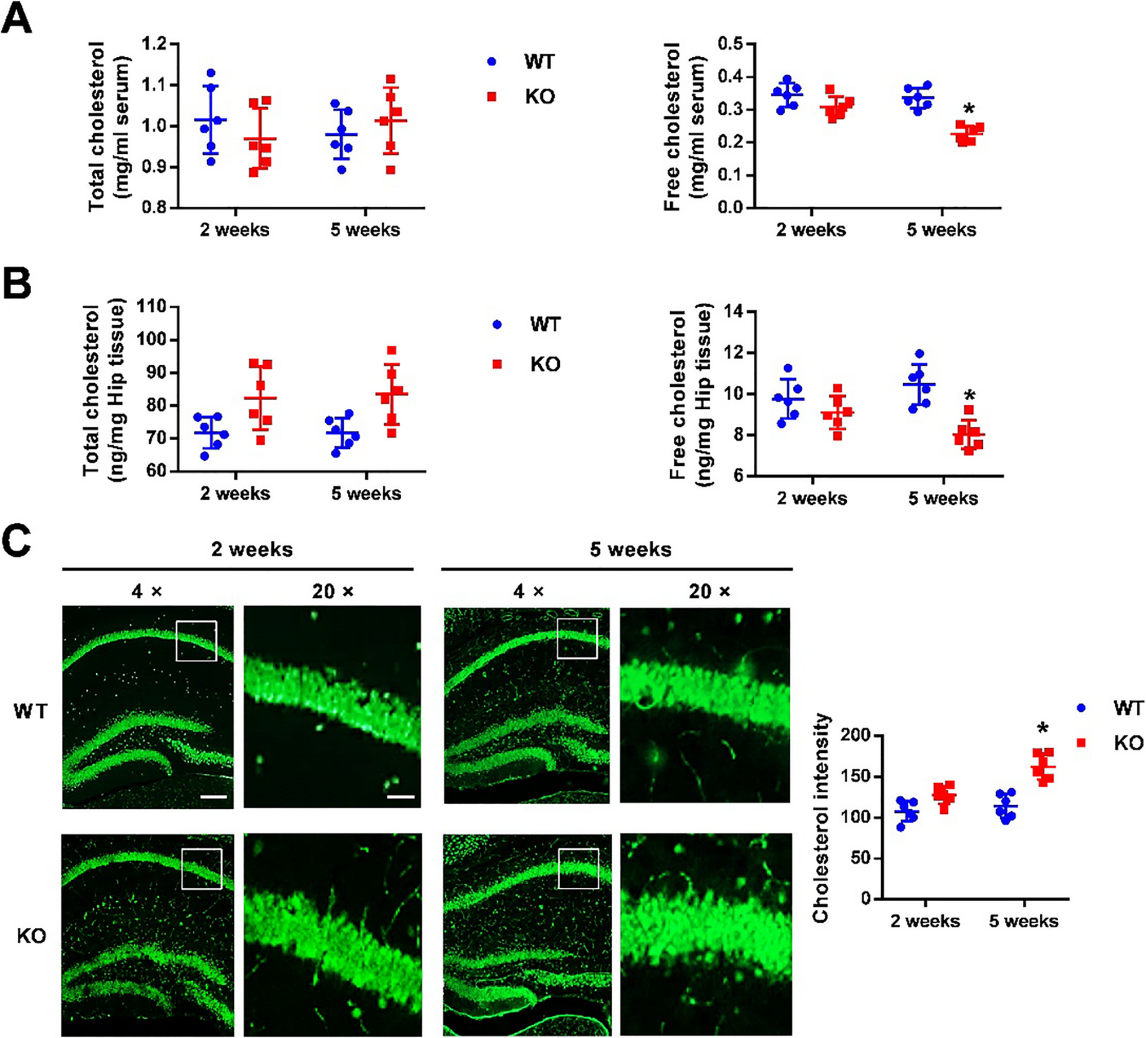
Excessive cholesterol is accumulated in the adolescent *Fmr1* KO hippocampus. **A, B** Levels of total cholesterol and free cholesterol were detected in serum (**A**) and hippocampus tissue (**B**) from WT and KO mice. Free cholesterol was reduced over 5 weeks in KO mice. *n* = 6 mice per group; * *p* < 0.05 versus WT. Cholesterol content was normalized with protein and expressed as ng/mg of tissue. *n* = 6 mice per group; * *p* < 0.05 versus WT, two-way ANOVA with Tukey’s multiple comparisons test. **C** Filipin III staining showed the cholesterol (green) in the hippocampal CA1 region under 4 × and 20 × magnification, respectively. Green represents positive neurons. The statistical results showed that the cholesterol intensity in CA1 region increased in 5-week-old KO mice. Left: scale bar = 1000 μm; Right: scale bar = 100 μm. *n* = 6 mice per group; * *p* < 0.05 versus WT, two-way ANOVA with Tukey’s multiple comparisons test

Cholesterol is highly concentrated in caveolae domains. Recent findings indicate that Cav1 directly binds to cholesterol, and this protein-lipid interaction is thought to be essential for caveolae formation [32, 33]. We determined cholesterol contents by quantitative measurements and filipin III staining. Total cholesterol levels in the serum or hippocampus were the same in both 2-week- and 5-week-old WT and KO mice, but free cholesterol levels were significantly lower in 5-week-old KO mice than in WT mice (Fig. 6A, B). Namely, cholesterol ester was increased in 5-week-old KO mice, indicating the accumulation of excessive cholesterol in *Fmr1* KO mice. Consistently, filipin III staining also showed increased cholesterol concentrations in the CA1 region of 5-week-old KO mice (Fig. 6C). These data support the notion that excessive cholesterol accumulation in the hippocampus promotes caveolae formation, facilitating GluA2 internalization under certain circumstances.

### Mβ-CD Restores Abnormal GluA2 Trafficking and Behaviors in *Fmr1* KO Mice

**Fig. 7:**
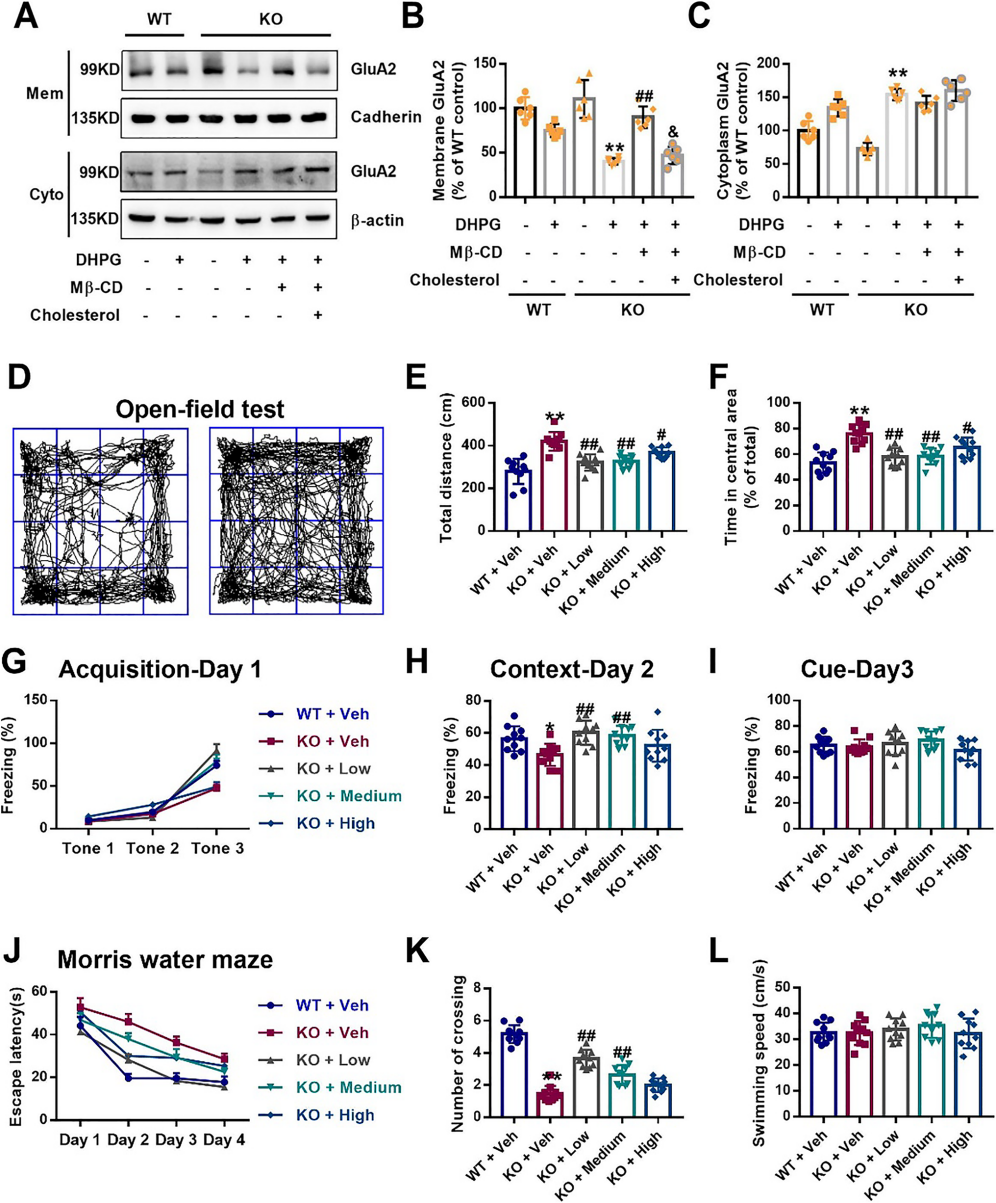
Mβ-CD reverses DHPG-induced GluA2 trafficking and abnormal behaviors of adolescent *Fmr1* KO mice. **A** Immunoblots detected GluA2 levels on membranes and in the cytoplasm of hippocampal slices, with pan-cadherin and β-actin as internal control, respectively. **B** Mβ-CD enhanced the surface level in the KO hippocampus upon DHPG treatment, but the effect was reversed by cholesterol. *n* = 6 mice per group; ** *p* < 0.01 versus KO control; ## *p* < 0.01 versus KO DHPG alone; & *p* < 0.05 versus KO DHPG and Mβ-CD, one-way ANOVA with Tukey’s multiple comparisons test. **C** The cytoplasmic GluA2 level was slightly decreased under Mβ-CD, although there was no significant difference. *n* = 6 mice per group; ** *p* < 0.01 versus KO control, one-way ANOVA with Tukey's multiple comparisons test. **D** Sample traces of locomotor activity in the open field test. **E, F** *Fmr1* KO mice subcutaneously injected with low (125 mg/kg), medium (250 mg/kg), and high (500 mg/kg) doses of Mβ-CD for 2 weeks showed a reduction in the total distance travelled (**E**) and time spent in center area (**F**). *n* = 10 mice per group; ** *p* < 0.01 versus WT mice; ^#^ *p* < 0.05, ^##^ *p* < 0.01 versus KO mice. **G** On training day 1, mice were exposed to three tone-foot shock pairs. KO mice with Mβ-CD exhibited increased freezing during the last phase. **H** Low and medium doses of Mβ-CD improved contextual fear learning in KO mice. **I** Mβ-CD had no effect on cued fear learning.* n* = 10 mice per group; * *p* < 0.05 versus WT mice; ^##^ *p* < 0.01 versus KO mice alone. **J** In the Morris water maze test, KO mice injected with Mβ-CD showed reduced escape latency, with an increase in learning days. **K** Low and medium doses of Mβ-CD enhanced the number of crossings in KO mice. **L** No significant difference between groups was observed in swimming speed.* n* = 10 mice per group; ** *p* < 0.01 versus WT mice; ^##^ *p* < 0.01 versus KO mice alone, one-way ANOVA with Tukey’s multiple comparisons test **(E–L)**

To assess the role of Cav1-mediated cholesterol and membrane/lipid rafts in DHPG-triggered AMPA receptor internalization, we used the cholesterol-binding agent methyl-β-cyclodextrin (Mβ-CD), which depletes cell surface cholesterol [34]. The results revealed that Mβ-CD (5 mM, 60 min) prevented DHPG-induced excessive GluA2 endocytosis in the KO hippocampus, with higher membrane and lower cytoplasm levels than DHPG alone (Fig. 7A–C). In addition, cholesterol (15 μg/ml, 60 min) reversed the effects of Mβ-CD on GluA2 endocytosis (Fig. 7A–C). These data indicate that DHPG-induced GluA2 trafficking depends on the structural integrity of caveolae in neurons.

Based on the preliminary results, different doses of Mβ-CD (125, 250, 500 mg/kg) were finally used to test whether Mβ-CD affects abnormal behaviors in *Fmr1* KO mice. Locomotor activity was analyzed in an open-field test to characterize hyperactivity, a common phenotype in FXS [35]. We found that all three doses of Mβ-CD significantly reduced the total distance traveled and the time spent in the central area in KO mice (Fig. 7D–F). In the fear conditioning paradigm described previously [25], we subjected mice to three pairs of CS-US during the learning phase on day 1 (Fig. 7G). During the acquisition period, KO mice injected with Mβ-CD exhibited higher responses than control mice (Fig. 7G). On day 2, both low and medium doses of Mβ-CD significantly increased freezing times during the hippocampus-dependent contextual recall test (Fig. 7H), whereas no significant differences between groups were observed during the amygdala-dependent cued fear condition on day 3 **(**Fig. 7I). Because spatial working memory is hippocampus-dependent [36], we next determined the effect of Mβ-CD on impaired spatial memory in KO mice tested on the Morris water maze. After Mβ-CD injection, KO mice were able to quickly find the platform on the following day, while vehicle-treated KO mice took until the fourth day to find it (Fig. 7J). Both low and medium doses of Mβ-CD dramatically increased the number of platform crossings in KO mice in the Morris water maze test (Fig. 7K), and these effects had nothing to do with swimming speed (Fig. 7L). Taken together, these findings show that Mβ-CD can alleviate the behavioral defects of *Fmr1* KO mice, implying its potential clinical usage to treat FXS.

## Discussion

Although clathrin-dependent internalization of neurotransmitter receptors has been extensively documented, little is known about the contribution of caveolins to endocytosis in neurons. In this study, we showed that Cav1 is involved in mGluR-triggered AMPA receptor endocytosis within the LTD and obtained evidence for the mGluR theory of FXS in view of structural signal formation.

### mGluR- and NMDAR-LTD in the Different Stages of FXS

The mGluR theory hypothesizes that the psychiatric and neurological aspects of FXS are a consequence of exaggerated Group I mGluR activation [5]. Here, since FXS is a developmental retardation disease, we focused on the changes in mGluR-LTD in the *Fmr1* KO hippocampus at different developmental stages. Using the Group I mGluR agonist DHPG, we observed augmented hippocampal mGluR-LTD in *Fmr1* KO mice aged p28-p35 but not in those aged p8-p15. Consistently, Toft et al. [17] reported that with NMDAR blockers, only p30-p60 *Fmr1* KO mice exhibited more mGluR-LTD than WT mice, suggesting that prolonged mGluR signaling in FXS results from specific alterations during development. Although Cooke et al*.* [37] proposed that the enhancement of mGluR-LTD in *Fmr1* KO mice was due to a lack of amino acids in the incubation medium, we suggest that one of the important reasons is the differential protein expression that mediates AMPA receptor endocytosis. Caveolin-coated pits in the postsynaptic membranes of glutamatergic nerves are primarily found in the late stages of growth and development [38]. Additionally, Cav1 protein levels gradually increase after birth due to the inhibitory effect of FMRP on *Cav1* mRNA translation [11]. We therefore considered that Cav1 was not expressed high enough to initiate mGluR-triggered endocytosis during early development. Moreover, there was no difference in Cav1 levels between WT and KO mice at this stage, and mGluR-LTD was normal in infant *Fmr1* KO mice. However, Cav1 expression increased in adolescence, especially in *Fmr1* KO mice, which may have been involved in the excessive endocytosis of AMPARs and led to mGluR-LTD enhancement.

Because at early stages, mGluR-LTD is presynaptic and independent of protein synthesis [39], we propose another reason related to protein synthesis. At later stages, mGluR-LTD is a dendritic protein synthesis-dependent form of synaptic plasticity [40, 41]. Although increased protein synthesis is observed in multiple brain regions of FXS mouse models and in cells derived from FXS patients [42, 43], some studies have indicated that protein synthesis is not required for mGluR-LTD in *Fmr1* KO mice [27, 44]. Consistently, we observed that the ERK and mTOR signaling pathways involved in protein synthesis were not further activated by DHPG in the KO hippocampus. Thus, the enhancement of mGluR-LTD in *Fmr1* KO mice did not require protein synthesis, perhaps because the “LTD protein” levels were elevated due to excessive translation during development. Our study suggests Cav1 as one kind of LTD protein whose levels are increased in the adolescent *Fmr1* KO hippocampus and whose expression is no longer induced by DHPG.

### Role of Caveolins in Synaptic Plasticity

Caveolae are 50 ~ 100-nm flask-shaped invaginations of the plasma membrane and are identified by the presence of the protein caveolin, which is essential for their biogenesis [45]. Caveolae harbor numerous signaling proteins, largely through their interaction with caveolin. They have been hypothesized to act as central hubs for intracellular signaling, facilitating signal transduction or, in some cases, sequestering and limiting activation of signaling cascades [45, 46]. This raises the possibility that caveolae might be involved in synapse formation and plasticity. Head et al*.* [47] demonstrated that neuron-targeted Cav1 improved NMDA- and BDNF-mediated signaling and enhanced dendritic growth. The resultant structural alterations in hippocampal neurons are associated with improvements in hippocampal-dependent learning and memory [25]. Another study has shown that Cav1 can also directly interact with and regulate the function of Group I mGluRs in the hippocampus and facilitate mGluR1 and mGluR5 expression [14]. Cav1 may thus positively regulate the transport of mGluR1/5 to the membrane surface and then amplify mGluR signaling, which may be responsible for the exaggerated mGluR in FXS. Accordingly, we previously reported that overexpressed Cav1 in *Fmr1* KO mice interfered with synaptic facilitation and LTP formation in the ACC [11].

Here, we demonstrate that Cav1 is involved in AMPAR endocytosis in the *Fmr1* KO hippocampus. Cav1 interference reversed mGluR-LTD and decreased the mGluR-induced reduction in surface AMPARs. Conversely, Cav1 overexpression promoted AMPAR endocytosis from lipid rafts. Additionally, other studies have provided evidence for the localization of GluA2 to caveolae-like structures in hippocampal neurons [48, 49], which structurally supports the participation of Cav1 in GluA2 trafficking. However, we cannot exclude the role of clathrin in normal mGluR-LTD because DHPG is still able to induce LTD in the hippocampal slices of *Cav1* KO mice, although at a smaller magnitude than in WT controls [15]. As DHPG promptly induced Cav1 expression, we suggest that Cav1-mediated GluA2 endocytosis is primarily dependent on mGluR1/5 activity and therefore attribute the enhanced mGluR-LTD in the *Fmr1* KO hippocampus to excessive Cav1 levels.

### Potential of Cyclodextrin in the Treatment of FXS

Cav1 is an integral membrane protein required for the formation of cholesterol-enriched membrane microdomain caveolae. Recent findings indicate that transport of newly synthesized cholesterol from the endoplasmic reticulum to the plasma membrane is mediated by caveolin proteins [50, 51]. In addition, Cav1 directly binds cholesterol, and cholesterol is required for the insertion of recombinant Cav1 into lipid membranes [32, 52]. Cav1 expression is increased in several neurodegenerative diseases, including Alzheimer’s disease (AD), suggesting Cav1 as a potential therapeutic target [53]. Our studies found that Cav1 expression in *Fmr1* KO mice is increased due to the loss of FMRP translation repression, implying the relevance of Cav1 for FXS pathogenesis [11]. Previous reports have shown that compounds from herbs, such as curcumin, daidzein, and salidroside, decrease Cav1 expression and/or phosphorylation [54, 55]. However, none has a specific effect on caveolae. Some studies have used cyclodextrin (CD), a cholesterol scavenger, to directly prevent caveolae formation [56]. 2-Hydroxypropyl-β-cyclodextrin (HP-β-CD) treatment has been evaluated in the AD mouse model and found to reduce the neurotoxic effects of Aβ [57]. Additionally, CD-based therapies have demonstrated promising advances with HP-β-CD in clinical trials for Niemann-Pick and Tangier disease [58]. Here, we found elevated cholesterol ester contents in the *Fmr1* KO hippocampus accompanied by alterations in Cav1 expression. These findings provide new insights into the altered lipid profiles in FXS. Although hypocholesterolemia was observed in a French-Canadian FXS population [59], this may have been due to the inconsistency between cholesterol metabolism in the blood and brain or to differences in age. Therefore, further investigations are warranted to better understand the association between cholesterol metabolism and FXS. A recent clinical study supported the clinical significance of lipid raft cholesterol alterations in FXS [60]. It is clear that lovastatin, a drug widely prescribed to treat high cholesterol, can correct excess hippocampal ERK-mediated protein synthesis and prevent mGluR-induced epileptogenesis in *Fmr1* KO mice [61]. We therefore used Mβ-CD to restore GluA2-containing AMPA receptor endocytosis. Moreover, Mβ-CD administered subcutaneously twice a week for 2 weeks to *Fmr1* KO mice attenuated hyperactivity and rescued hippocampus-dependent contextual fear memory and spatial memory but did not affect amygdala-dependent cued fear memory. Although mGluR5 antagonists can also significantly improve synaptic development, plasticity, and behavioral abnormalities in *Fmr1* KO mice [62], clinical trials have thus far failed due to widespread adverse reactions. CD has been used as a drug delivery carrier because of its safety, so it is expected to be applied to regulate the function of mGluR in patients with FXS.

In conclusion, our study indicates that exaggerated mGluR-LTD in the *Fmr1* KO hippocampus is development-dependent and related to overexpressed Cav1 followed by AMPA receptor endocytosis under mGluR1/5 activation. Moreover, massive cholesterol accumulation contributes to redundant caveolae formation, which provides the platform for mGluR-triggered Cav1 coupling to GluA2. In addition, cholesterol depletion by Mβ-CD recovers AMPA receptor trafficking and ameliorates the principal behavioral disorders observed in *Fmr1* KO mice. Our findings elucidate the important role of Cav1 in the pathology of FXS from the perspective of mGluR theory and suggest a novel and safe strategy for the treatment of FXS.

## Supplementary Information

Below is the link to the electronic supplementary material.

Supplementary file1 (DOCX 1035 KB)

## Data Availability

The data that support the findings of the present study are available from the corresponding author upon reasonable request.
