# Supplementary material for: Caveolin-1-Mediated Cholesterol Accumulation Contributes to Exaggerated mGluR-Dependent Long-Term Depression and Impaired Cognition in Fmr1 Knockout Mice
Source: Mol Neurobiol. 2023 Mar 1;60(6):3379–95. doi: 10.1007/s12035-023-03269-z (PMC10122623; doi:10.1007/s12035-023-03269-z)
Supplement: Supplementary file 1 — Supplementary file1 (DOCX 1035 KB) [file 12035_2023_3269_MOESM1_ESM.docx]

**Supplementary information (Molecular Neurobiology)**

**Title**

Caveolin-1 mediated cholesterol accumulation contributes to exaggerated mGluR-dependent long-term depression and impaired cognition in *Fmr1* knockout mice

**Authors**

Li Luo^1#^, Le Yang^1#^, Kun Zhang^1#^, Shi-Meng Zhou^2^, Yan Wang^3^, Liu-Kun Yang^2^, Bin Feng^4^, Shui-Bing Liu^2^, Yu-Mei Wu^2^, Ming-Gao Zhao^1^, Qi Yang^1^*

**Affiliation**

### ^1^Precision Pharmacy & Drug Development Center, Department of Pharmacy, Tangdu Hospital, Fourth Military Medical University, Xi’an 710038, China

### ^2^Department of Pharmacology, School of Pharmacy, Fourth Military Medical University, Xi’an 710032, China

### ^3^Department of Gastroenterology and Endoscopy Center, No.986 Hospital, Fourth Military Medical University, Xi’an 710054, China

^4^State Key Laboratory of Military Stomatology, National Clinical Research Center for Oral Diseases, Shaanxi International Joint Research Center for Oral Diseases, Department of Pharmacy, School of Stomatology, Fourth Military Medical University, Xi’an 710054, China

^#^ These authors contributed equally to this work

***Corresponding author**

Qi Yang, Precision Pharmacy & Drug Development Center, Department of Pharmacy, Tangdu Hospital, Fourth Military Medical University, Xi’an 710038, China

ORCID: 0000-0001-8959-1342

Phone: 0086-29-84777154; Fax: 0086-29-84777763; Email: [yangqifmmu@126.com](mailto:yangqifmmu@126.com)

**Figure S1**


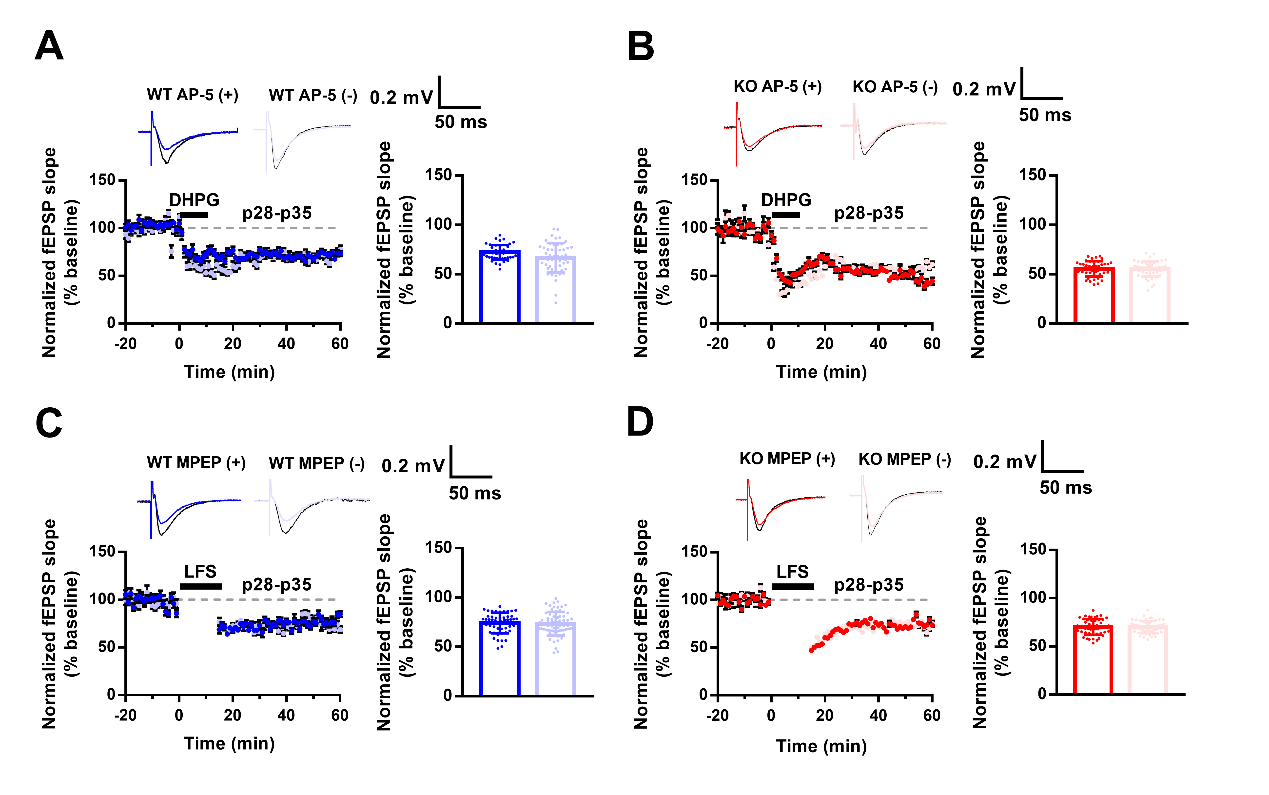


**Figure S1.** **Application of AP5 or MPEP had no effect on DHPG induced mGluR-LTD and LFS induced NMDAR-LTD. (A, B)** Induction of mGluR-LTD by bath application of DHPG (100 μM, 10 min) at age p28-p35 in WT **(A)** and *Fmr1* KO mice **(B)** with or without AP5 (50 μM, 30 min). *n* = 56 channels recorded from 8 slices of five WT mice, *n* = 63 channels recorded from 9 slices of six KO mice, unpaired two-tailed Student’s t test. **(C, D)** NMDAR-LTD was induced by LFS (900 pulses at 1 Hz, 15 min) at age p28-p35 in WT **(C)** and *Fmr1* KO mice **(D)** with or without MPEP (40 μM, 30 min). *n* = 61 channels recorded from 9 slices of five WT mice, *n* = 73 channels recorded from 9 slices of six KO mice, unpaired two-tailed Student’s t test. **Top**, Representative traces of 64 channels recorded at baseline (black) and 1 h after stimulation (colors). Calibration bars: 0.2 mV, 50 ms. **Bottom left**, Normalized fEPSP slope (% baseline) of LTD from total active channels. **Bottom right**, Summary graph of the final average fEPSP slope during the last 30 min.

**Figure S2**


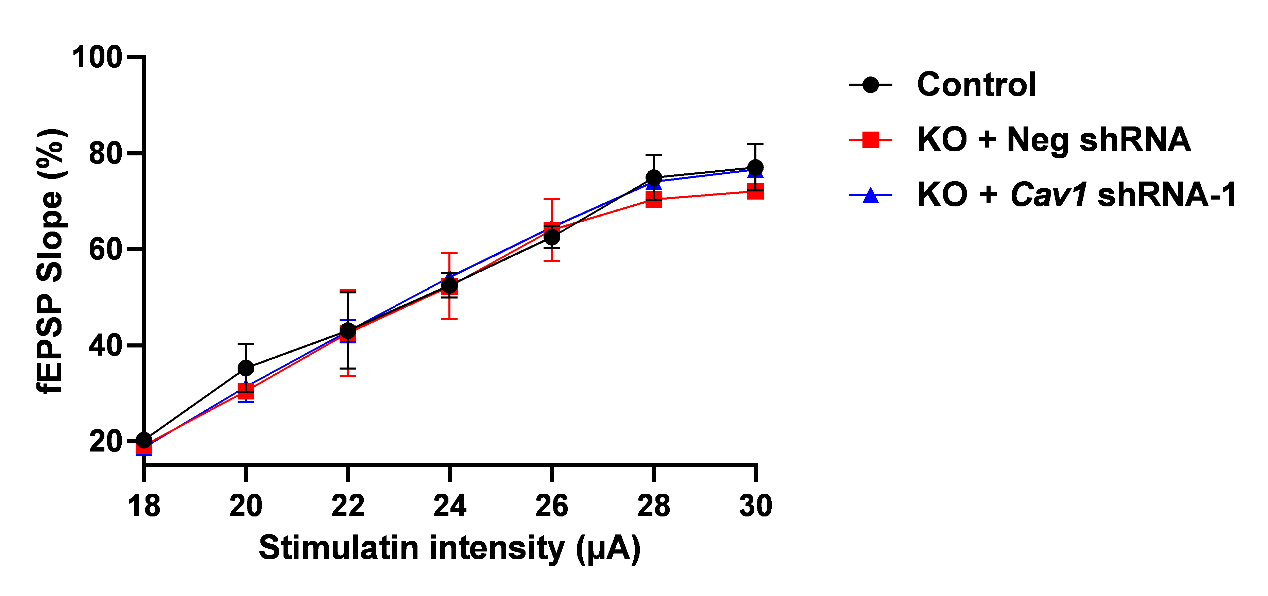


**Figure S2.** **I/O curve making by fEPSP slope versus stimulation intensity.** Plot of input-output curves showed that there was no difference in function synaptic transmission between KO + Vehicle shRNA, KO + *Cav1* shRNA-1 and Control group in hippocampus. n = 6 neurons/3 mice.

**Figure S3**


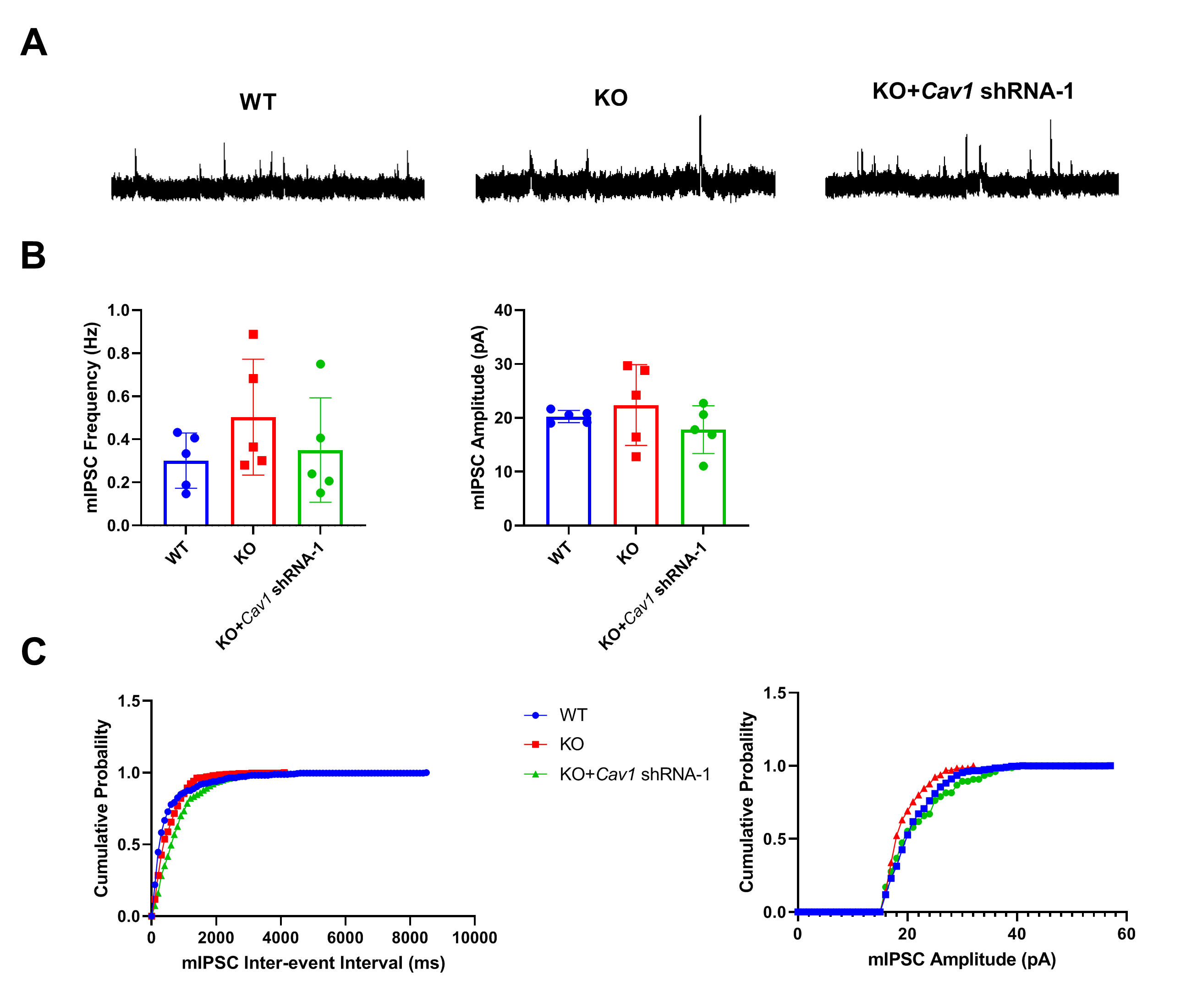


**Figure S3.** **Knockdown of Cav1 had no effect on hippocampal inhibitory transmission.** **(A)** Representative mIPSC recorded in hippocampal neurons with a holding potential of +10 mV. **(B)** Histograms of cumulative frequency (left) and amplitude (right) of mIPSC in cells of each group, n = 5 mice per group, unpaired two-tailed Student’s t test. **(C)** Quantitative analysis of neurons in each group of hippocampus showed that Cav1 in *Fmr1* KO mice did not affect the frequency (left) and amplitude (right) of mIPSC (n =11 neurons from 4 mice).

**Figure S4**


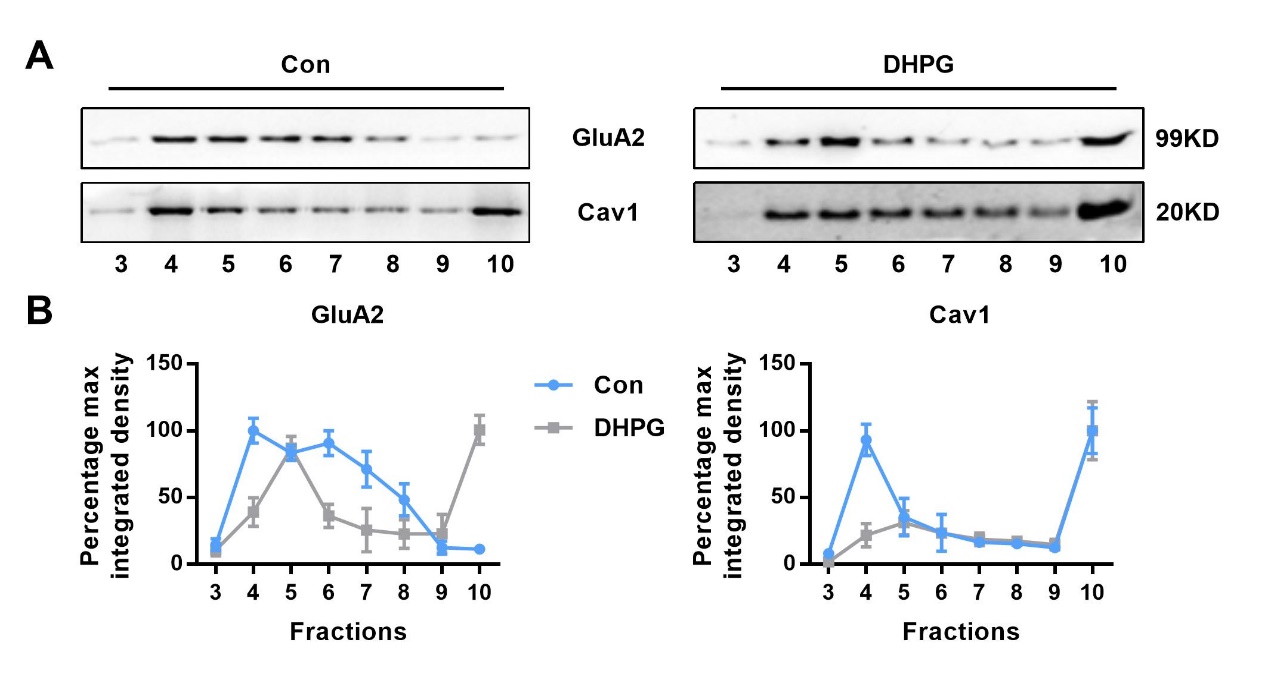


**Figure S4. DHPG-induced GluA2 endocytosis depends on caveolae. (A)** Cav1 was present in both the lighter caveolae fractions (4 to 5) and in cytoplasmic lysate (heavier fraction 10) of control group. By contrast, incubation with DHPG resulted in the detection of GluA2 in both caveolae and the cytoplasm, accompanied by enhanced Cav1 levels in all fractions. *n* = 4 dishes in each group. **(B)** A graphical representation of integrated densities across fractions for each condition for GluA2 and Cav1.
